# Nanoparticle labeling identifies slow cycling human endometrial stromal cells

**DOI:** 10.1186/scrt473

**Published:** 2014-07-04

**Authors:** Lina Xiang, Rachel W S Chan, Ernest H Y Ng, William S B Yeung

**Affiliations:** 1 Department of Obstetrics and Gynaecology, The University of Hong Kong, Pokfulam, Hong Kong, SAR, China; 2 Centre of Reproduction, Development of Growth, LKS Faculty of Medicine, The University of Hong Kong, Pokfulam, Hong Kong, SAR, China

## Abstract

**Introduction:**

Evidence suggests that the human endometrium contains stem or progenitor cells that are responsible for its remarkable regenerative capability. A common property of somatic stem cells is their quiescent state. It remains unclear whether slow-cycling cells exist in the human endometrium. We hypothesized that the human endometrium contains a subset of slow-cycling cells with somatic stem cell properties. Here, we established an *in vitro* stem cell assay to isolate human endometrial-derived mesenchymal stem-like cells (eMSC).

**Methods:**

Single-cell stromal cultures were initially labeled with fluorescent nanoparticles and a small population of fluorescent persistent cells (FPC) remained after culture of 21 days. Two populations of stromal cells, namely FPC and non-FPC were sorted.

**Results:**

Quantitative analysis of functional assays demonstrated that the FPC had higher colony forming ability, underwent more rounds of self-renewal and had greater enrichment of phenotypically defined prospective eMSC markers: CD146^+^/CD140b^+^ and W5C5^+^ than the non-FPC. They also differentiate into multiple mesenchymal lineages and the expression of lineage specific markers was lower than that of non-FPC. The FPC exhibit low proliferation activities. A proliferation dynamics study revealed that more FPC had a prolonged G_1_ phase.

**Conclusions:**

With this study we present an efficient method to label and isolate slow-proliferating cells obtained from human endometrial stromal cultures without genetic modifications. The FPC population could be easily maintained *in vitro* and are of interest for tissue-repair and engineering perspectives. In summary, nanoparticle labeling is a promising tool for the identification of putative somatic stem or progenitor cells when their surface markers are undefined.

## Introduction

Somatic tissues are comprised of connective tissue or stromal components, and mesenchymal stem cells of the stroma are believed to be responsible for tissue regeneration and remodeling [1]. The inner mucosal lining of the uterus is the endometrium, which consists of epithelial and mesenchymal stromal cells. The endometrium displays remarkable regenerative capacity during the reproductive years of a woman [2]. Stem/progenitor cells residing in the lower basalis layer of the endometrium are believed to be responsible for the cyclic growth after menstruation [3]. Recently, subpopulations of the endometrial stromal cells have been shown to exhibit properties of mesenchymal stem cells [4,5]. Human bone marrow-derived cells can also incorporate into the endometrium in low numbers [6,7]. Therefore, there is an emerging concept that the human endometrial-derived mesenchymal stem-like cells (eMSC) are responsible for the cyclical regeneration of the human endometrium [5].

Somatic stem cells are characterized by their dual abilities to self-renew and to differentiate into progenitors of various lineages [8]. During differentiation, somatic stem cells divide asymmetrically to give rise to two daughter cells with different cell fates [9]. One daughter cell is a copy of the original stem cell and continues to function as a stem cell, while the other differentiates, divides and gives rise to mature cells. It is commonly accepted that somatic stem cells are usually quiescent in nature [10]. Their infrequent division would prevent them from exhaustion during tissue regeneration and repair. However, rapidly regenerating tissues indicate that quiescence may not be an obligatory stem cell feature [11,12]. Currently, there is no study on the cycling kinetics of endometrial stem cells.

We hypothesize that eMSC are slow-cycling. In mouse endometrium, the existence of slow-cycling stem-like cells has been reported [13-15]. However, traditional tracking tools for human tissue stem cells without definitive cell surface markers cannot isolate candidate somatic stem cells for functional investigation. To overcome this obstacle we developed a method for the isolation of eMSC based on their slow-cycling property. In this study, we use fluorescent nanoparticles for tracking slow-cycling cells in a live heterogeneous population of endometrial stromal cells. The fluorescence of the nanoparticles is bio-stable and photo-stable. Therefore, it can be tracked even after a prolonged period of culture without perturbing cellular function [16]. In addition, these nanoparticles are randomly distributed among daughter cells without altering the differentiation potential of the stem cells [17]. We postulated that the loaded nanoparticles would persist in the slow-cycling cells (fluorescent persistent cells, FPC) but dilute to an undetectable level in the actively-proliferating cells (non-FPC), such as progenies of eMSC. Here, we report the successful use of nanoparticles for the isolation of a subset of endometrial-derived mesenchymal stromal cells with somatic stem cell properties. In the initial part of this study, we compared the fluorescence signals in the endometrial stromal cells after cultivation for 15 and 21 days to determine the optimal dilution time for cells to undergo sufficient rounds of cell division. In the later part of the study, post-labeled cells cultivated for 21 days were used for isolation and characterization of the endometrial stromal FPC.

## Methods

### Human tissues

Human endometrial tissue was collected from 19 ovulating women, 35- to 49-years old undergoing hysterectomy for non-endometrial pathologies, who had not taken hormonal therapy for three months before surgery. Ethical approval was obtained from the Institutional Review Board of the University of Hong Kong/Hospital Authority Hong Kong West Cluster. Informed written consent was obtained from each patient. The stage of the menstrual cycle was categorized into proliferative (n = 7) and secretory (n = 12) by experienced histopathologists based on hematoxylin-eosin-stained endometrial sections. The histological criteria for endometrial dating of the menstrual phase were according to Noyes *et al.*[18].

### Preparation of single cell suspensions of human endometrial stromal cells

The endometrium was scraped off from the underlying myometrium, minced and digested in PBS containing collagenase type I (300 μg/mL, Sigma-Aldrich, St Louis, MA, USA) and deoxyribonuclease type I (40 μg/mL, Roche Diagnostics, Mannheim, Germany) for one hour at 37°C, as described previously [19]. Ficoll-Paque (GE Healthcare, Uppsala Sweden) density-gradient centrifugation was used to remove red blood cells. Endometrial stromal cells were selected using negative selection with epithelial marker anti-EpCAM antibody-coated Dynabeads (Clone BerEP4, Invitrogen, Grand Island, NY, USA) and elimination of leukocytes using anti-CD45 antibody-coated Dynabeads (Invitrogen).

### Qtracker® labeling and tracking

A portion of the freshly purified endometrial stromal cell suspension was loaded with commercially available nanoparticles (Qtracker® 655 cell labeling kit, Invitrogen), while the other portion of cells was used a control. The reagents in the kit use a targeting peptide to deliver red-fluorescent nanoparticles into the cytoplasm of live cells. Qtracker® reagent A and B were premixed following the manufacture’s instruction and incubated with one million stromal cells containing fresh growth medium, DMEM-F12 medium (Sigma-Aldrich) containing 10% FBS (Gibco, Grand Island, NY, USA), 1% antibiotics (Gibco) and 2 mmol/L glutamine (Gibco). The cells were incubated with the dye solution for 45 minutes at 37°C, after which cells were washed twice with growth medium. About 2 × 10^5^ cells were then plated into 100-mm petri dishes (BD Biosciences, San Jose, CA, USA) coated with gelatin. Cells were cultured in growth medium at 37°C in a humidified carbon dioxide incubator. Medium was changed every seven days.

Regular monitoring of the Qtracker® dye in the stromal cell culture was conducted every three to four days using an Eclipse TE300 inverted microscope (Nikon, Tokyo, Japan). The petri dish containing the cultured cells was divided into ten areas and images of the cells within the areas were acquired with the Image-Pro-Plus 6.0 software (Media Cybernetics, Warrendale, PA, USA). The Qtracker® fluorescence labeling indexes after culturing for 0, 1, 3, 6, 8, 15 and 21 days were determined by dividing the number of fluorescence^+^ cells by the total number of cells counted. Four plates of cells from each day were counted.

### Fluorescence-activated cell sorting analysis

Endometrial stromal cells labeled with the Qtracker® dye were cultured for 15 and 21 days and fluorescence-activated cell sorting (FACS) was performed in the University of Hong Kong Core Facility with a FACSAria I flow cytometer (BD Biosciences) using the FACSDIVA software (BD Biosciences). The cells were selected with electronic gating according to the forward and the side scatter profiles (Additional file 1: Figure S1A and S1B) using the BD diva software (BD Biosciences). Freshly labeled cells from the same patient were used as the positive control (Additional file 1: Figure S1C), while the unlabeled cells were used as the negative control (Additional file 1: Figure S1D). Cells were incubated with propidium iodide (PI) to exclude non-viable cells. Based on their fluorescence intensities, FPC and non-FPC were sorted (Additional file 1: Figure S1E). The purity of each sample was > 90%. FACS data were analyzed using the FlowJo software (Tree Star, Ashland, OR, USA).

### Colony initiating cell assay

For assessment of colony-forming ability, FACS sorted endometrial stromal FPC and non-FPC from post-labeled day 15 (D15) and day 21 (D21) were plated at a clonal density of 50 cells/cm^2^ onto gelatin coated 100-mm petri dishes and cultured for 15 days. Cells were incubated at 37°C in 5% carbon dioxide with the culture medium changed every seven days. Regular monitoring of the cells was performed under an inverted microscope (Nikon) to identify colonies derived from single cells. Colony-forming units (CFU) were stained with toluidine blue (Sigma-Aldrich) on day 15. Large CFU were defined as colonies with > 4,000 cells and small CFU were those with < 4,000 cells [19]. The colony forming ability was determined by the number of CFU formed divided by the number of cells seeded, multiplied by 100.

#### *In vitro* serial cloning

Individual large and small CFU from passage 1 (P1) were trypsinzed using cloning rings (Sigma-Aldrich) to determine the self-renewal capacity of cells from endometrial stromal FPC and non-FPC. Colonies collected from post-labeled D15 and D21 were assessed. The cell number of each CFU was determined and the cells were re-seeded onto gelatin-coated dishes at a density of 20 cells/cm^2^ and cultured for a further 15 days for formation of clones in the secondary passage (P2). This process continued until the cells could no longer form CFU.

### Fluorescence-activated cell sorting analyses of endometrial mesenchymal stem-like cell marker expression

The expression level of prospective endometrial mesenchymal stem cell markers (co-expression of CD146/CD140b and W5C5) on endometrial stromal cells post-label D21 (P0) were analyzed using multicolor flow cytometry. Dissociated cells were re-suspended at a concentration of 2 × 10^6^ cells/ml in 0.1% BSA/PBS and incubated with antibodies against fluorescein isothiocyanate (FITC)-conjugated anti-CD146 (1 mg/ml, P1H12 clone, mouse immunoglobulin G1 (IgG1), Abcam, Cambridge, UK), phycoerythrin (PE)-conjugated anti-platelet-derived growth factor beta (PDGFRβ) (CD140b, 2.5 μg/ml, PR7212 clone, mouse IgG1, R&D Systems, Minneapolis, MN, USA) and PE-conjugated anti-mesenchymal stem cell (W5C5, mouse IgG1, Biolegend, San Diego, CA, USA) in the dark for 45 minutes on ice. Isotype matched controls were included for each antibody. The cells were then washed with 0.1% BSA/PBS three times (five minutes each) and kept in 200 μl of 0.1% BSA/PBS for flow cytometric analysis using the BD FACS Aria I (BD Biosciences). Gating setting of fluorescence label cells, negative and positive controls of stromal cells were prepared as described above.

### Total proliferative potential

The proliferative potential of CFU derived from the endometrial stromal FPC and the non-FPC were examined by separately pooling 10 to 15 large CFU and 20 to 30 small CFU, then expanding them in culture seeding at 2,000 cells/cm^2^ in triplicates of six-well plates. Only chase D21 P1 cells were used. They were passaged every eight to twelve days when the cultures reached 70% to 90% confluence. The process was continued until senescence of the cells. The numbers of cells at each passage were determined.

### Determination of proliferation activity

#### Trypan blue exclusion assay

Clonally derived cells from D21 P2 were used to determine the proliferation activity of the endometrial stromal FPC and the non-FPC. Unselected stromal cells from the same passage were used as control. Cells were seeded at 50 cells/cm^2^ in triplicates of gelatin coated 12-well plates and cultured for 15 days. Cell growth was photographed under an inverted microscope. Cell viability was determined by the trypan blue exclusion assay. Triplicate wells of viable cells for each population of cells were counted on a hemocytometer after trypsinization.

#### DNA proliferation assay

Clonally derived cells from D21 P2 FPC and non-FPC were cultured for 15 days. The DNA content of the cell was measured using the CyQUANT® NF Cell Proliferation Assay (Invitrogen). Briefly, the culture medium was removed from triplicates of 12-well plates and 500 μl of the dye solution was added into each well. The plate was incubated at 37°C for 60 minutes and the absorbance unit (AU) was measured on a microplate reader (Tecan, Männedorf, Switzerland) with an excitation wavelength of 485 nm and an emission wavelength of 530 nm.

### Generation of Cdt1-expressing cells

To visualize the G_1_ phase of the cell cycle in living cells, single clonally derived FPC and non-FPC from D21 chase P2 from three patient samples were plated in six-well plates (2 × 10^4^ cells) and transduced with Premo™ CdC 10 dependent transcript 1 red fluorescent protein reagent (Cdt1-RFP, Invitrogen) according to the manufacturer’s instructions. HeLa and unselected endometrial stromal cells from P2 were used as controls for the analysis. HeLa cells were grown in DMEM supplemented with 10% fetal bovine serum (FBS) and 1% penicillin. Endometrial stromal cells were cultured in the standard conditions described above.

### Time lapse imaging of live Cdt1-expressing cells

To capture single Cdt1-expressing cells, high-speed spinning disk wide field imaging was performed at the University of Hong Kong Core Facility on a PerkinElmer system (PerkinElmer Life and Analytical Sciences, Waltham, MA, USA) at 18 hours post-fluorescence labeled. The microscope stage incubation chamber was maintained at 37°C. Phase-contrast and fluorescence images were recorded at 15-minute intervals. To quantify the duration of the G_1_ phase of individual Cdt1-expressing cells, the intensity of the nuclear red fluorescence was determined within a period of 42 hours. Cells that exhibited a gradual increase in the fluorescence intensity to a maximum followed by a gradual fading within the recording period were selected for analysis. The length of the G_1_ phase was measured as the time interval between the first frame with the appearance of the fluorescence to the last frame with the disappearance of the fluorescence within the recording period (Additional file 2: Figure S2A). Cdt1-expressing cells that displayed the following patterns were not included for analysis: continuous fluorescence throughout the recording period (Additional file 2: Figure S2B), fluorescence at the beginning of the recording (Additional file 2: Figure S2C), or fluorescence still observed at the end of the recording (Additional file 2: Figure S2D). Image analysis was performed by using the Image Pro Plus software (Media Cybernetics, Rockville, MD, USA). The time lapse recording of Cdt1-transduced HeLa cells revealed a G_1_ duration (6.03 ± 3.5 hour, a total of n = 20/cell from three experiments) similar to a previous report [20].

### Mesenchymal lineage differentiation assay

#### In vitro cell culture

The multipotency of endometrial stromal FPC and non-FPC was tested *in vitro* using clonally derived cells from D21 P2 chase. Large CFU were expanded in normal culture conditions until 80% confluence. Cells were trypsinized, re-seeded in triplicate at a seeding density of 200 cells/cm^2^ in six-well plates and cultured in either adipogenic, osteogenic or myogenic induction medium for four weeks as outlined in Additional file 3: Table S1 [21]. For chondrogenic differentiation, 5 × 10^5^ cells were centrifuged as a micromass and cultured in chondrogenic induction medium for four weeks. Some of the endometrial stromal cells were cultured in serum-containing medium for four weeks and served as the undifferentiated control.

#### Histochemical and immunohistochemical staining

Assessment of multipotency on differentiated cells was confirmed using histochemical stains: Oil Red O (Sigma-Aldrich), safranin-O or immunohistochemical stains using antibodies against peroxisome proliferation activated receptor γ (PPARγ), alpha smooth muscle actin (αSMA) (Dako Cytomation, Glostrup, Denmark), osteopontin (Abcam), and collagen type II (Abcam) for adipogenic, myogenic, osteogenic and chondrogenic differentiation, respectively, as outlined in Additional file 3: Table S1. After four weeks of culture in the respective induction medium, the cells were fixed in 4% paraformaldehyde and blocked with 10% serum (Sigma-Aldrich) to the host species of the secondary antibody for 30 minutes. Primary antibodies (Additional file 4: Table S2) or isotype matched control antibodies were incubated overnight at 4°C followed by the corresponding secondary antibodies (Additional file 4: Table S2) for one hour. Cells were then incubated with the avidin biotin complex reagent (Vector Laboratories, Burlingame, CA, USA) for 30 minutes and positive immunoreactivities were visualized using 3, 3'-diaminobenzidine (DAB) substrate solution (Sigma-Aldrich) counterstained with hematoxylin. All incubations were performed at room temperature unless otherwise specified and washes with PBS were conducted between each step. The cells were examined under an Axioskop microscope (Zeiss, Oberkochen, Germany) and images were acquired with a Photometric CoolSNAP charge-coupled device camera (Roper Scientific, Tucson, AZ, USA) using the CoolSNAP version 1.1 software.

#### Western blot analysis

Proteins from *in vitro* differentiated FPC and non-FPC were extracted with cell lysis buffer (Ambion, Grand Island, NY, USA). The protein samples (5 μg) were mixed with 5X SDS loading buffer (60 mM Tris–HCl pH 6.8, 2% SDS, 0.1% bromophenol blue, 25% glycerol and 14.4 mM β-mercaptoethanol) and denatured at 95°C for 10 minutes. Protein samples were subjected to sodium dodecyl sulfate-polyacrylamide gel electrophoresis, and transferred to polyvinylidene difluoride (PVDF) membranes. Membranes were blocked with 5% skim milk (Nestle, Vevey, Switzerland) in PBS containing 0.1% Tween-20 (PBST) for 30 minutes and incubated with primary antibodies at appropriate concentrations (Additional file 5: Table S3) overnight at 4°C. The membranes were stained with appropriate horseradish peroxidase-conjugated secondary antibodies (Additional file 5: Table S3) for one hour at room temperature. The protein bands were visualized by enhanced luminal-based chemiluminescence (Westsave UP™; AbFrontier, Seoul, Korea). Endometrial stromal cells cultured in serum containing medium were used as the control. Non-immune immunoglobulin of the same isotype as the primary antibody was used as negative control. The scanned Western blot bands were quantified densitometrically and the values were normalized to the amount of β actin using Image J software (US National Institutes of Health, USA).

### Quantitative real-time polymerase chain reaction

Quantitative real-time polymerase chain reaction (qPCR) with Taqman probes was used to examine mesenchymal lineage and pluripotent genes. Total RNA was extracted with the Absolutely RNA RT-PCR microprep kit (Agilent Technologies, Santa Clara, CA, USA). The quality and quantity of the total RNA was checked by spectrophotometry. The RNA was reverse transcribed by the high capacity complementary DNA reverse transcription kit (Roche Applied Science, Basel, Switzerland). Taqman probes (Roche Applied Biosystems) were used to quantify expression of the lineage (Additional file 3: Table S1) and pluripotent (Additional file 6: Table S4) genes. Real-time PCR was performed with a 7500 Real-Time PCR System (Applied Biosystems, Grand Island, NY, USA) using the following parameters: 2 minutes at 50°C, 10 minutes at 95°C, then 40 cycles of 15 seconds at 95°C and 1 minute at 60°C. The results are presented as relative gene expression compared with internal control 18S using the 2-ΔΔC_t_ method [22]. Determination was made in triplicate from three separate samples.

### Statistical analysis

Data were analyzed using the GraphPad PRISM software (version 5; GraphPad Software Inc., La Jolla, CA, USA). All experiments were performed using at least three different patient samples. The D’Agostino-Pearson test determined that the data were not normally distributed. Non-parametric Kruskal-Wallis one-way analysis of variance followed by Mann Whitney was used to analyze the data. *P* < 0.05 was considered as significantly different. Data are expressed as means ± standard error of the mean (SEM).

## Results

### Nanoparticle-labeling identifies a population of endometrial stromal FPC

**Figure 1:**
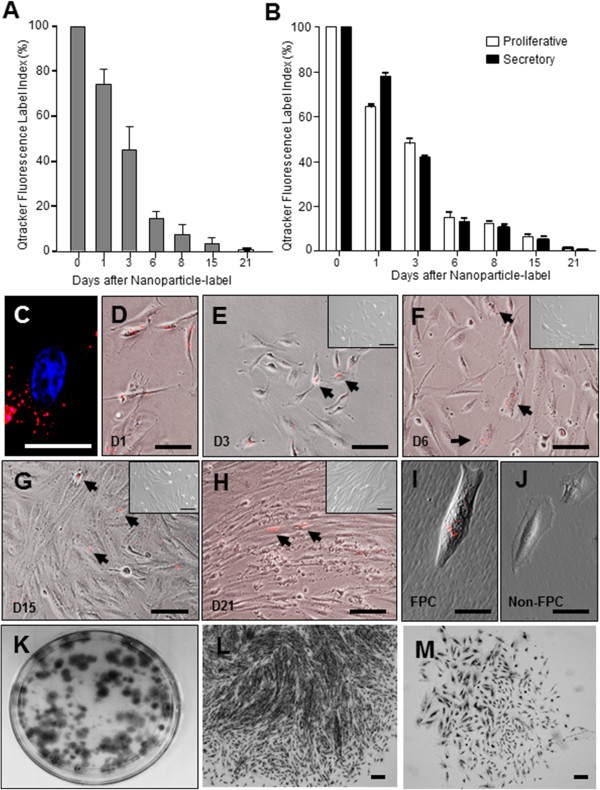
**Nanoparticle-labeled endometrial stromal cells.** Qtracker® label of endometrial stromal cells. Quantitation of nanoparticle-labeled endometrial stromal cells **(A)** with different duration of chase (n = 4 per time point) and **(B)** at different menstrual phases (n = 3, proliferative, white; n = 3, secretory, black). Fluorescence expressing cells are reported as means ± SEM of the percentage of total stromal cells seeded. Day 1 post-labeled stromal cells stained with DAPI nuclei stain (blue) indicating the nanoparticles (red) are located in the cytoplasm **(C)**. Representative phase contrast images of nanoparticle-labeled stromal cells at different days in culture **(D – H)**. Nanoparticle-labeled cells (arrows) detected on day 1 **(D)** and then among the unlabeled stromal cells on day 3 **(E)**, day 6 **(F)** and day 15 **(G)**. Fluorescent signal retained at day 21 **(H) (E - H)**. Negative controls of unlabeled stromal cells are shown in the insets. Representative photographs of post-labeled day 21 endometrial stromal cells after FACS analysis as FPC **(I)** and non-FPC **(J)** population in culture for five days. Culture dish displaying distribution of stromal colony forming units (CFU) **(K)** after 15 days of culture. Morphology of large **(L)** and small **(M)** CFU. Scale bars = 50 μm **(C, I, J)** and 100 μm **(D - H, L - M)**. DAPI, 4',6-diamidino-2-phenylindole; FACS, fluorescence-activated cell sorting; SEM, standard error of the mean.

Freshly isolated endometrial stromal cells at different menstrual phases were loaded with the Qtracker® dye. The distribution and pattern of the fluorescence signal in the cells were assessed for 21 days. All the cells showed 100% intracellular punctuate red fluorescent signal at 24-hour post-labeling and the nanoparticles accumulated in the perinuclear cytoplasm (Figure 1A, C, D). The nanoparticles did not contribute to adverse effects on proliferation or viability of post-labeled cells (data not included). The percentage of cells declined rapidly to 3.6 ± 0.8% over 15 days of culture (Figure 1A, E, F, G). Only 0.8 ± 0.4% of the endometrial stromal cells retained the fluorescence on D21 post-labeling (Figure 1A, H). These stromal cells retaining the fluorescence are termed FPC. The temporal change in the fluorescence labeling index was similar in the endometrial stromal cells from the proliferative and the secretory phases (Figure 1B). Based on these results, chase periods of 15 and 21 days were selected for comparison of the efficiency in obtaining an enriched population of slow-proliferating cells from the culture.

### Clonogenicity and self-renewal of endometrial stromal FPC and non-FPC

Qtracker® loaded endometrial stromal cells were isolated using FACS after D15 and D21 chases to generate the FPC (Figure 1I) and the non-FPC (Figure 1J) populations. The cell morphology of the two populations in culture was similar. Single FPC and non-FPC were then subjected to the *in vitro* colony forming assay. Figure 1K shows the formation of clones after 15 days in culture, when clonogenic clones can be depicted [19]. CFU were categorized into large CFU > 4,000 cells (Figure 1L) and small CFU < 4,000 cells (Figure 1M).

**Figure 2:**
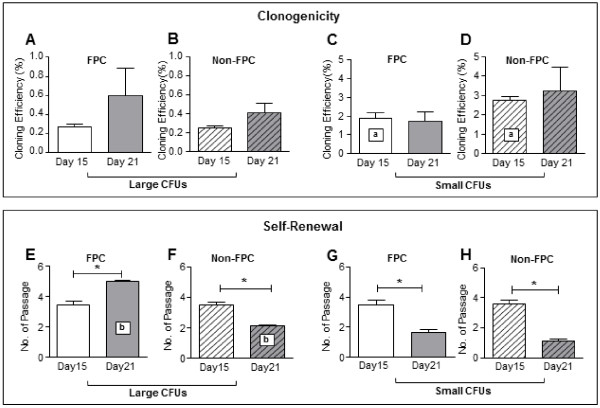
**Clonogenicity and self- renewal ability of stromal FPC and non-FPC post-labeled day 15 and 21. (A – D)** Cloning efficiency of endometrial stromal cells post-labeled with nanoparticles at day 15 (white bars) and 21 (grey bars). Cloning efficiency of large CFU **(A)** FPC and **(B)** non-FPC. Cloning efficiency of small CFU **(C)** FPC and **(D)** non-FPC. **(E – H)** Self-renewal activity of endometrial stromal cells post-labeled with nanoparticles at day 15 (white bars) and 21 (grey bars) using serial cloning assay. Large CFU self-renewal ability of **(E)** FPC and **(F)** non-FPC. Small CFU self-renewal ability of **(G)** FPC and **(H)** non-FPC Results reported as means ± SEM, n = 4, *^, a, b^*P* <0.05. CFU, colony-forming units; FPC, fluorescent persistent cells; SEM, standard error of the mean.

The cloning efficiency was determined for the large and small CFU from both populations. The percentage of FPC that formed large CFU was 0.3 ± 0.1% (n = 4) at chase-D15 and 0.6 ± 0.2% (n = 4) at chase-D21 (Figure 2A). Although there was a trend of higher large CFU forming ability with increased duration of chase, the increase did not reach statistical significance (*P* = 0.34). The corresponding cloning efficiencies of the non-FPC large CFU were 0.3 ± 0.1% and 0.4 ± 0.1%, respectively (Figure 2B). The cloning efficiencies for FPC small CFU at chase-D15 (1.9 ± 0.3%) and chase-D21 (1.7 ± 0.5%) were similar (Figure 2C). The cloning efficiencies of non-FPC small CFU were also similar (D15: 2.7 ± 0.2%; D21: 3.2 ± 1.2%; Figure 2D). There was no statistical difference in the formation of small CFU for FPC (*P* = 0.69) and non-FPC (*P* = 1.00) at the two chase periods.

The cloning efficiency of non-FPC small CFU at chase-D15 was higher (2.7 ± 0.2%, Figure 2D) than FPC (1.9 ± 2.3, *P* <0.05, Figure 2C). There was no statistical difference in the formation of large CFU for FPC and non-FPC at chase D15 (*P* = 0.62). There was also no differences between the two populations for large (*P* = 0.58) and small (*P* = 0.68) CFU at chase D21.

A better assessment of the somatic stem cell characteristic was to quantify the self-renewal abilities of the clonally derived cells *in vitro*. A total of four individual large and small CFU per patient sample (n = 4) obtained from the clonogenic assays were used. FPC large CFU at chase-D21 underwent significantly more rounds of self-renewal (5.0 ± 0.1) than those at chase-D15 (3.5 ± 0.3, *P* <0.05, Figure 2E). In contrast, non-FPC large CFU at chase-D15 underwent more rounds of self-renewal (3.5 ± 0.2) than those at chase-D21 (2.2 ± 0.1, *P* <0.05, Figure 2F). For small CFU formed by FPC or non-FPC, the self-renewal activities were significantly higher for cells at chase-D15 (3.5 ± 0.3 and 3.5 ± 0.2, respectively) than those at chase-D21 (1.6 ± 0.2 and 2.1 ± 0.1, respectively, *P* <0.05, Figure 2G, 2H).

The self-renewal activity for FPC large CFU at chase-D21 was higher (5.0 ± 0.1%, Figure 2E) than non-FPC (2.2 ± 0.1, *P* <0.05, Figure 2F), while the self-renewal ability was similar between FPC and non-FPC at chase–D15: large CFU (*P* = 0.89) and small CFU (*P* = 0.87) and chase-D21 small CFU (*P* = 0.09).

Since FPC at chase-D21 displayed greater self-renewal ability, they were used in subsequent experiments.

### Endometrial stromal FPC exhibit greater self-renewal and clonogenic activities

**Figure 3:**
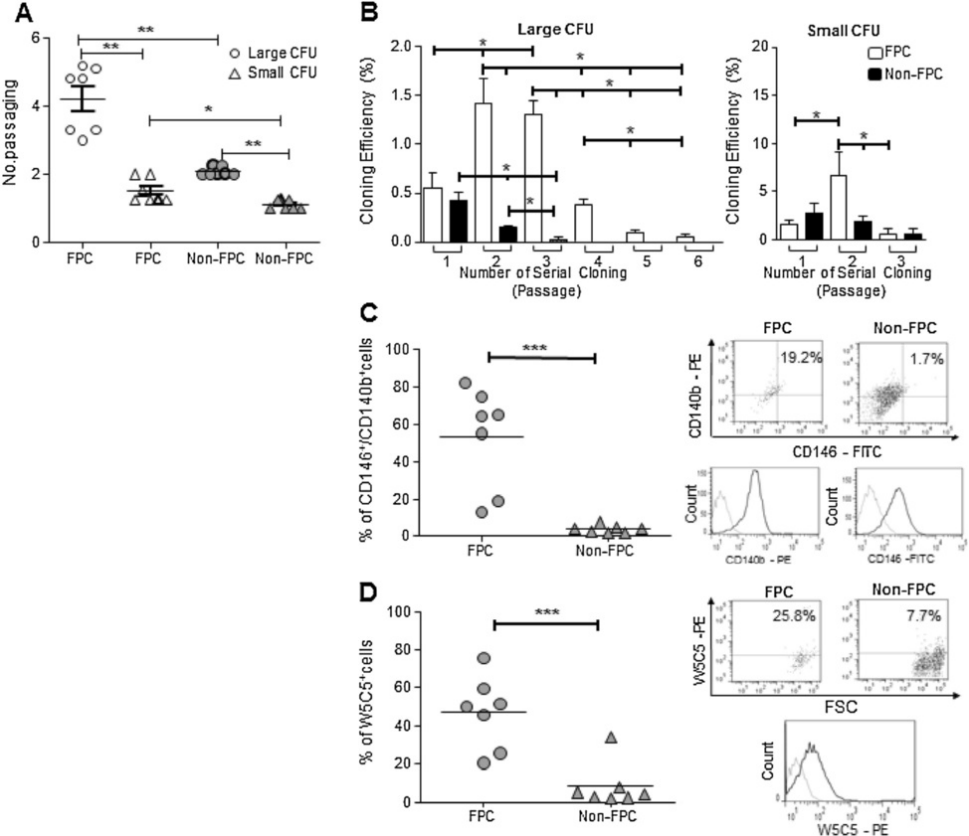
**Serial passage, clonogenicity and phenotyping of endometrial FPC and non-FPC post labeled day 21. (A)** Rate of serial passage is shown for both large and small CFU of FPC (white) and non-FPC (black). **(B)** Percentage of large and small CFU at each passage of serial cloning for FPC (white) and non-FPC (black). Percentage of **(C)** CD146^+^/CD140b^+^ and **(D)** W5C5^+^ cells from FPC and non-FPC populations. Bar represents the mean. Representative dot-plots for co-staining of CD146/CD140b and single staining of W5C5. Single parameter histograms for individual markers CD146-FITC, CD140b-PE and W5C5-PE. Grey line indicates background fluorescence with isotype matched IgG control Results are reported as mean ± SEM, n = 7, *P < 0.05, **P < 0.01, ***P < 0.001. CFU, colony-forming units; FPC, fluorescent persistent cells; SEM, standard error of the mean.

The self-renewal ability of endometrial stromal FPC large CFU at chase-D21 (4.2 ± 0.4) was significantly higher than that of the non-FPC large CFU (2.1 ± 0.1, *P* < 0.01, n = 7; Figure 3A) when more samples were analyzed. The self-renewal activity was also higher for the endometrial stromal FPC small CFU (1.50 ± 0.13) than for the non-FPC small CFU (1.1 ± 0.1, *P* < 0.05).

Next, we studied the sub-cloning characteristics of the two populations post-label D21. In general, only 0.90 ± 0.09% (n = 19) of the endometrial stromal cells were nanoparticle-labeled after 21 days. FPC large CFU formed clones in the first six rounds of recloning and stopped thereafter (Figure 3B). Interestingly, significantly higher cloning efficiencies of the endometrial stromal FPC large CFU were observed in the secondary (P2) and tertiary (P3) subcloning when compared with that of the primary cloning (P1, Figure 3B, *P* <0.05). These findings suggest that the freshly isolated FPC population is enriched with stem/progenitor cells that divide symmetrically to produce more stem/progenitor cells resulting in higher cloning efficiency in P2 and P3. The derived stem/progenitor cells subsequently produce transit amplifying (TA) cells with gradually decreasing proliferation potential at each serial cloning step. In contrast, the non-FPC large CFU exhibited limited recloning ability with cloning efficiency that declined significantly (Figure 3B, *P* < 0.05) in P2 and P3. Small CFU of both FPC and non-FPC also displayed limited cloning ability after three rounds of recloning (Figure 3B).

### Prospective eMSC markers are expressed on endometrial stromal FPC

Previous reports showed the existence of a phenotypically distinct and relatively rare population of eMSC expressing CD146^+^/CD140b^+^ and W5C5^+^[4,23]. The expression of these two eMSC markers in the endometrial stromal FPC and non-FPC after D21 chase were analyzed by flow cytometry (Figure 3C, 3D). The percentage of cells co-expressing CD146^+^/CD140b^+^ was significantly higher in the endometrial FPC population (53.2 ± 10.1%, n = 7) than in the non-FPC population (3.7 ± 0.8%, *P* < 0.001). The proportion of cells expressing the marker W5C5^+^ was 47.0 ± 7.2% (n = 7) for the FPC and 8.3 ± 4.4% for the non-FPC; the two values were significantly different (*P* < 0.01). The enrichment of these reported markers in the FPC population indicates that these cells are enriched for phenotypically defined eMSC.

### Passaging efficiency of endometrial stromal FPC

The proliferative potential was determined by serial passaging of the clonally derived FPC and non-FPC until senescence. The number of cells generated by a particular population and the cumulative cell output are shown in Table 1. The yield from a single large CFU of endometrial stromal FPC ranged from 1.3 × 10^11^ to 2.4 × 10^11^ (167 to 244 days, n = 3), while that from a non-FPC ranged from 6.6 × 10^8^ to 1.4 × 10^9^ (66 to 115 days, n = 3). The total time required for the large CFU to reach senescence was 217.6 ± 25.4 days for the endometrial stromal FPC and 98.3 ± 16.2 days for the non-FPC. For the small CFU of endometrial stromal FPC, the cell yield was from 2.4 × 10^6^ to 4.5 × 10^6^ and grew for 57 ± 9.5 days (n = 3). The corresponding values for non-FPC ranged from 1.5 × 10^6^ to 3.7 × 10^6^ (30 to 34 days, n = 3) before reaching senescence at 36.3 ± 4.5 days. There was no significant difference in the cell yield between large or small CFU for FPC (*P* = 0.10) or non-FPC (*P* = 0.10).

**Table 1:** Total cell output from human stromal FPC and non-FPC CFU

**Cell type**	**CFU size**	**Cell yield**	**Length of experiment (days)**
**FPC**	Large	1.8 ± 0.3 × 10^11^	217.7 ± 25.4
	Small	3.4 ± 0.6 × 10^6^	57 ± 9.5
**Non-FPC**	Large	9.5 ± 0.3 × 10^8^	98.3 ± 16.2
	Small	2.4 ± 0.6 × 10^6^	36.3 ± 4.5

Number of patient samples (n = 3) from 10 to 15 large and 20 to 30 small individual stromal CFU were serially passaged until senescence. Results are reported as means ± SEM. CFU, colony-forming units; FPC, fluorescent persistent cells; SEM, standard error of the mean.

### Endometrial stromal FPC have low proliferation activities

**Figure 4:**
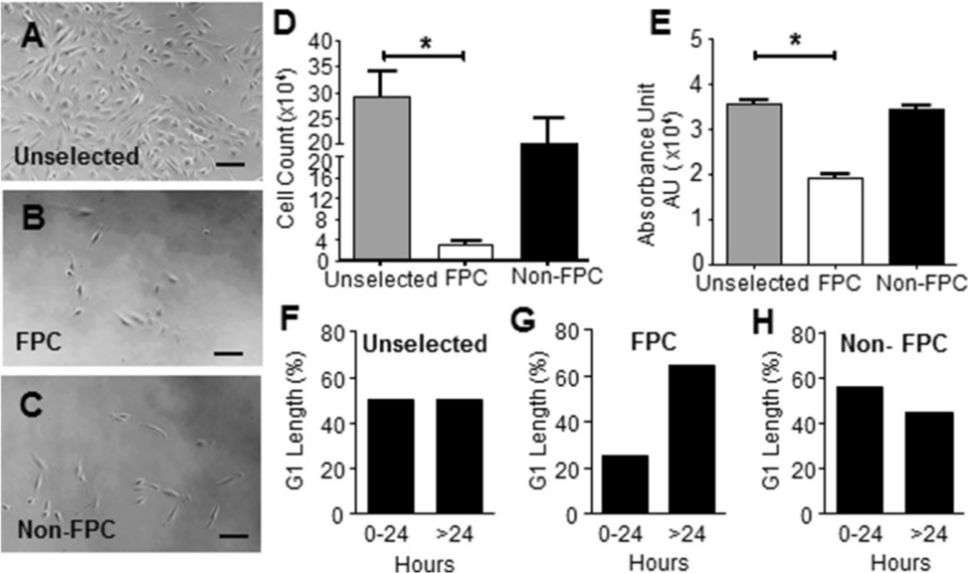
**The cell proliferation activity (A - E) and G**_**1**_**-phase length (F - H) of human endometrial stromal FPC and non-FPC.** Representative photographs of **(A)** unselected stromal cells, **(B)** FPC and **(C)** non-FPC morphology in the proliferation assay at day 5. Cell proliferation assessment on unselected endometrial stromal cells (grey), clonally derived FPC (white) and non-FPC (black) after 15 days in culture using **(D)** trypan blue assay and **(E)** CyQUANT NF cell proliferation assay. The absorbance unit (AU) was measured at a wavelength of 480 nm with a reference wavelength of 530 nm. Scale bar: 100 μM. Cells were obtained from the same patient. Results are reported as means ± SEM, n = 3, *P <0.05. Quantification of the G_1_ length in Cdt-1-expressing stromal cells of FPC and non-FPC populations followed by time-lapse microscopy within a period of 42 hours. The distribution of G_1_-phase for **(F)** unselected stromal cells (n = 20 cells), **(G)** FPC (n = 20 cells) and **(H)** non-FPC (n = 18 cells) from three patient samples. FPC, fluorescent persistent cells; SEM, standard error of the mean.

The proliferative activity was assessed using two assays. By day 5, more unselected endometrial stromal cells were depicted under the microscope than FPC (Figure 4B) and non-FPC (Figure 4C) populations. This was supported by the quantitative trypan blue assay (Figure 4D), which showed remarkably higher mean cell count for the unselected endometrial stromal cells (29.26 × 10^4^ ± 4.64 × 10^4^) than the clonally derived FPC on day 15 (2.96 × 10^4^ ± 7.41 × 10^4^, *P* <0.05, n = 3). The mean cell count for the endometrial stromal non-FPC was 20.27 × 10^4^ ± 0.74 × 10^4^ and was not different from that of the unselected endometrial stromal cells but higher than that of FPC. A similar finding was obtained with the fluorescence-based assay by measuring the DNA content of the cultured cells (Figure 4E). The absorbance was significantly higher for the unselected endometrial stromal cells (3.58 × 10^4^ ± 0.18 × 10^4^ AU) than the FPC (1.94 × 10^4^ ± 0.43 × 10^4^ AU, *P* < 0.05, n = 3). No difference was found between the absorbance for the endometrial stromal non-FPC (3.43 × 10^4^ ± 0.61 × 10^4^ AU) and the unselected endometrial stromal cells. Collectively, these findings together with the passaging efficiency demonstrate the enrichment of slow-proliferating cells in the endometrial stromal FPC subset.

### G_1_ phase lengthening in the endometrial stromal FPC

We next examined the length of the G_1_ phase in the different cell populations using a fluorescence unbiquitylation-based cell cycle indicator, Cdt1. The expression of Cdt1 is highest in cultured cells in the G_1_ phase [20]. The expression of Cdt1 in the unselected endometrial stromal cells, clonally derived FPC and non-FPC, was monitored at the single cell level for 42 hours. We analyzed only the Cdt1-expressing cells that showed a gradual increase in red fluorescence (entry of G_1_ phase) followed by a loss of the fluorescence (transit to the G_1_/S phase, Additional file 2: Figure S2A) within the recording period. The endometrial stromal cells have a doubling time of 24 hours [24]. Here, we compared the percentage of cells with a G_1_ phase longer or shorter than 24 hours in the studied cell populations. The length of the G_1_ phase in the endometrial stromal populations showed a wide distribution. For the unselected stromal cells, 50% of them had a G_1_ phase > 24 hours (Figure 4F, n = 20/cell). The proportion of FPC having a G_1_ phase > 24 hours (65%, Figure 4G, n = 20/cell) was higher than that of the non-FPC (44%, Figure 4H, n = 18/cell). Their difference did not reach statistical significance, probably due to the small sample size. The slow proliferation activity of the FPC may be associated with lengthening of the G_1_ phase.

### Endometrial stromal FPC have greater multipotency

eMSC can differentiate into mesenchymal lineages upon cultivation in specific induction media [4]. To determine the multipotent potential of the clonally derived-FPC and non-FPC, they were induced to differentiate into mesenchymal lineages *in vitro*.

**Figure 5:**
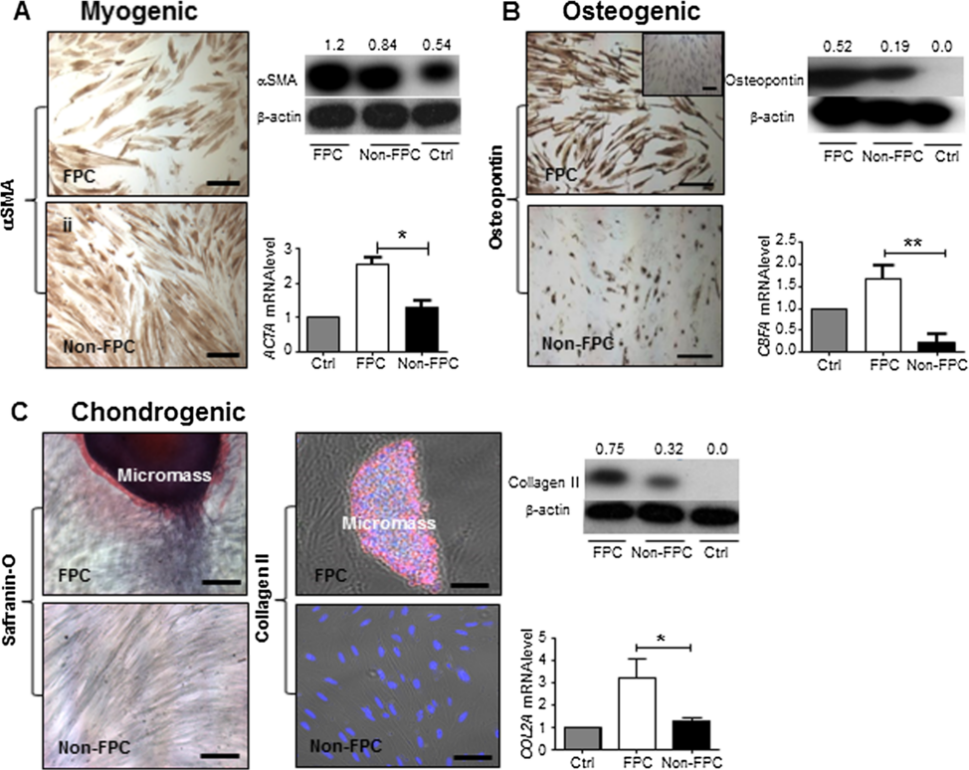
**Differentiation potential of human endometrial stromal FPC and non-FPC into mesenchymal lineages *in vitro*.** Myogenic differentiation **(A)** with αSMA (brown) staining on cells clonally derived from stromal large CFU of FPC and non-FPC. Protein expression and quantification of αSMA. Relative gene expression level of *ACTA* by real time PCR. Osteogenic differentiation **(B)** with osteopontin (brown) staining on cells clonally derived from stromal large CFU of FPC and non-FPC. Protein bands and quantification expression of osteopontin. Relative gene expression level of *CBFA1* by real-time PCR. Chondrogenic differentiation **(C)** with safranin-O (red) histochemical staining on cells clonally derived from stromal large CFU of FPC and non-FPC. Immunofluorescent staining with DAPI (blue) and collagen II (pink) on stromal large CFU of FPC and non-FPC. Micromass structure depicted from FPC chondrogenic induced cells. Protein bands and quantification expression of collagen II. Relative gene expression level of *COL2A1* by real-time PCR. mRNA expression levels were normalized to 18S. Expression of the control was set as one. Control cells stained for lineage markers shown in inset and western blots are unselected stromal cells grown in culture medium with fetal bovine serum for four weeks. Scale bar: 200 μm, including inset. Results shown from a single sample representative of three patients. Results are reported as means ± SEM for western blotting (n = 3) and for real-time PCR (n = 6), *P < 0.05, **P < 0.01. αSMA (*ACTA*), alpha smooth muscle actin; CBFA1: core binding factor 1; CFU, colony-forming unit; COL2A1: collagen type II alpha 1; FPC, fluorescent persistent cells; SEM, standard error of the mean.

Both populations of stromal cells showed strong protein expression of the early smooth muscle cell marker (Figure 5A) αSMA in immunohistochemical staining and in western blotting when cultured in the myogenic inducing medium. Quantitative analysis revealed a significantly higher mRNA expression of αSMA (ACTA) in the FPC than in the non-FPC (*P* < 0.05, Figure 5A).

For osteogenic differentiation (Figure 5B), both populations of stromal cells stained positive for osteopontin upon induction and its protein expression level was also detected by western blotting. The expression of the early stage osteogenic transcription factor, core binding factor alpha1 (CBFA1), was significantly higher in the FPC than in the non-FPC (*P* < 0.01, Figure 5B).

In chondrogenesis-inducing medium, only the FPC formed micromass pellets while the non-FPC remained as a monolayer of cells (Figure 5C). Safranin O staining of the FPC-derived micromass revealed the presence of sulfated proteoglycan. The cells in the masses possessed collagen II protein as shown in immunohistochemical staining and western blotting. Quantitative analysis of the collagen II transcript showed higher expression in the FPC population (Figure 5C, *P* < 0.05).

For the adipogenic lineage, FPC and non-FPC did not develop lipid-like droplets after induction and failed to stain with oil red O (data not shown). Both populations of stromal cells did not stain for the key adipogenic transcription factor, peroxisome proliferation activated receptor γ (PPARγ), and its protein expression was also not detected by western blotting (data not shown). However, quantitative analysis of the transcription factor CCA-AT/Enhancer Binding Protein alpha (C/EBP-α) revealed significantly higher expression in the FPC population (Additional file 7: Figure S3, *P* < 0.05).

Based on these data, both populations of stromal cells can differentiate into three of the four mesenchymal lineages studied. In particular, the FPC are significantly more multipotent as reinforced by the quantitative analysis of the data, indicating this subset contains more uncommitted progenitor cells.

### Molecular characteristics of the endometrial stromal FPC and non-FPC

**Figure 6:**
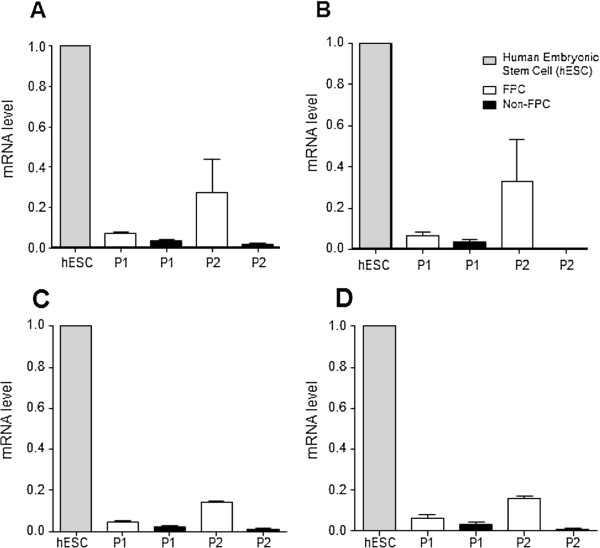
**Expression of pluripotent and self-renewal genes.** The relative gene expression levels of **(A)***NANOG*, **(B)***SOX2*, **(C)***OCT-4* and **(D)***BMI-1* of passage 1 and 2 (P1, P2) FPC (white bars) and non-FPC (black bars) in comparison to human embryonic stem cells (hESC, grey bars) as positive control. Results are reported as means ± SEM, n = 3. FPC, fluorescent persistent cells; SEM, standard error of the mean.

To gain further insight into the molecular properties of the two functionally distinct populations, we performed real-time PCR to determine their expression of genes associated with pluripotency in human embryonic stem cells (*NANOG*, *SOX2*, *OCT4*) [25], and self-renewal in somatic stem cells (*BMI-1*) [26]. Comparison of the FPC and non-FPC transcript expression at P1 revealed no differences for these genes (Figure 6). Interestingly, an up-regulation trend for the four genes (Figure 6A-D, n = 3) was observed in cells of the secondary FPC when compared to that of primary FPC and non-FPC, though the difference had not yet reached statistical significance (*P* = 0.10) probably because of the small sample size.

## Discussion

This study demonstrates the existence of a specific population of FPC in the human endometrial stromal culture. These cells exhibit stem cell characteristics, including high proliferation, self-renewal, differentiation potential and expression of prospective eMSC surface markers. In addition, the stromal FPC have slow-proliferating activity associated with lengthening of their G_1_ phase while non-FPC have restricted proliferative ability and a lack of specific markers associated with stemness, pluripotency suggesting that these cells are more differentiated cells and reaching replicative senescence. Many groups have used various assays, such as clonogenicity [19], side-population phenotype [27,28] and surface markers [4,23] to identify and characterize eMSC. However, to our knowledge there are no studies showing the existence of slow-cycling stem/progenitor cells in the human endometrium.

The classical approach of identifying slow-cycling somatic stem cells is using the bromouridine labeling technique, in which the stem cells retain the label after long term chase. However, the detection of the incorporated bromouridine requires fixation of the cells, making isolation of viable label-retaining cells for functional studies impossible. There is a need to develop a simple method for isolation of viable somatic stem/progenitor cells, which may be used for stem/progenitor cells without known surface markers. Here, we demonstrated that fluorescent nanoparticles can be used to identify and isolate viable endometrial cells with stem/progenitor cell properties. In pancreatic stem cells, the uptake of the fluorescent nanoparticles does not affect proliferation and gene expression, and the nanoparticles are transmitted equally to the daughter cells during cell divisions [17]. We have also tried to label the endometrial stromal cells with two other reported tracking dyes, namely PKH26 [29] and Vybrant CM-Dil™ [30], and found them unsuitable for the endometrial stromal cells; the PKH26 signal was lost after culturing for 21 days, while the Vybrant™ CM-Dil label became undetectable after trypsinization for flow cytometry analysis (unpublished data).

Several lines of evidence indicate FPC are candidate eMSC. Firstly, the endometrial stromal FPC have high efficiency in developing into colonies containing cells with proliferative potential higher than non-FPC. More importantly, cells from the FPC-derived large CFU can undergo substantial self-renewal, producing tertiary and higher order of clones. We consider the FPC from these large CFU as stem/progenitor cells, as they generate quaternary clones, defining the adult stem cell status with the ability to proliferate, self-renew and produce a large number of differentiated cells [31]. FPC-derived small CFU are initiated by TA cells, since they exhausted after several rounds of serial cloning. Thus, they were not fully characterized in this study.

Secondly, the clonogenic efficiency of the FPC increased whereas that of the non-FPC decreased in the secondary and tertiary passages. After isolation by flow cytometry (P1), the colony-forming ability of FPC was slightly higher than that of the non-FPC. A large significant variation between samples was observed, which may have masked any significant difference. The significant increase of clonogenic activity in the subsequent two passages (P2 and P3) was associated with an up-regulated expression of self-renewal and pluripotent genes. The cells derived from the large CFU of FPC displayed a much higher proliferation activity at clonal density after serial subcloning. Re-plating of the single FPC may trigger candidate eMSC to self-renewal exhibiting high proliferative potential and maintaining a durable capacity of generating progenies through expansion in subsequent passages. The nature of the trigger is not known, although micro-environmental niche is known to affect cell-fate decision, such as self-renewal versus differentiation [10,32]. Nonetheless, our current culture condition may not be optimal to sustain the stemness of FPC. Therefore, long-term *in vitro* culture of FPC leads to gradual loss in the proliferation potential, as shown by the stepwise reduction in the colony number with increase in passaging. A similar finding was also observed in small CFU of FPC at P2. These small CFU derived from TA cells displayed limited proliferative ability and quickly exhausted in later passages.

Thirdly, the endometrial stromal FPC demonstrated greater multipotent potential than the non-FPC. Although FPC could not fully differentiate into adipocytes because they fail to express the late adipogenic marker PPARγ [33], we did find both the FPC and non-FPC clones possessed the ability to develop into myogenic, osteogenic and chondrogenic lineage cells. Quantitative assays for expression of these lineage specific markers demonstrated that the FPC exhibited significantly greater multi-lineage potential than the non-FPC. The identification of candidate eMSC with high differentiation efficiency may be useful for future tissue engineering applications [34].

Fourthly, a high proportion of FPC expressed surface markers of eMSC. Studies reveal that human eMSC expressing the markers CD146^+^/CD140b^+^ and W5C5^+^ exhibit properties of bone marrow (BM) derived MSCs [3]. Endometrial stromal FPC are enriched with cells W5C5^+^ (47%) and CD146^+^/CD140b^+^ (53%). Given the perivascular localization of both these markers in the endometrium, it is highly likely that our FPC are BM derived. Consistent with the observed properties of FPC in this study, endometrial stromal CD146^+^/CD140b^+^ or W5C5^+^ populations also have high colony forming ability and multi-lineage potentials. The overall cloning efficiency (CE) of FPC found in our study was three-fold lower than that reported for the CD146^+^/CD140b^+^ cells [4]. However, we noticed that the CFU size for CD146^+^/CD140b^+^ clones was 10-fold lower than that in our study. The difference in the definition of CFU size may account for the lower cloning efficiency observed in stromal FPC. The discovery of the single marker W5C5, or recently identified as SUSD2, offers a simpler method for the isolation of the prospective eMSC [35]. Comparative studies have shown higher cloning efficiency for the magnetic beaded W5C5^+^ cells than the FACS sorted W5C5^+^ cells [23]. Unpublished observations from our group revealed high variation in the cloning efficiency of W5C5^+^ cells among human endometrial samples. Furthermore, the duration for clonogenic assessment of the W5C5^+^ cells *in vitro* was two to three times longer and the sizes of the colonies were smaller than those in the present study. Clonal studies have shown the endometrial stromal cell proliferation potential (clonogenic size) correlates with cell potency, both in healthy and pathologic conditions [3]. It remains to be determined whether these eMSC subsets represent different intermediate developmental stages of the same stem/progenitor cells or independent cell lineages which have originated from different stem/progenitor cell populations.

In this study, we used Cdt1-RFP to determine the length of the G_1_-phase in single cells, and found that a high proportion (65%) of FPC have a prolonged G_1_ phase. This finding is similar to the percentage of CD146^+^/CD140b^+^ and W5C5^+^ cells in the FPC population. Human placenta derived-MSCs [36] and brain parenchymal progenitors [37] also display a long G_1_ phase. A lengthening of the G1 cell cycle in the murine embryonic and neural stem cells correlates with differentiation [38,39]. On the other hand, a prolonged G_1_ transit is a property of mouse fetal liver hematopoietic stem cells [40] and brain parenchymal progenitors [37]. Available evidence suggests the environmental niche contributes to cell regulation in somatic stem cells. Hyaluronan maintains the slow-cycling property of MSCs by prolonging the G_1_[36]. The expression of hyaluronan synthase in human endometrium can potentially be responsible for the hyaluronan accumulation in slow cycling stromal cells [41]. In contrast, wound injury induces the brain parenchyma progenitors to enter the cell cycle, shortening the normally long G_1_ phase [37].

Slow-cycling and or quiescent state is important for maintaining the stem cell function of somatic stem cells. Continuous proliferation results in exhaustion of the stem cell function [32]. Our findings show that the slow cycling property of the endometrial stromal FPC does not affect their stem cell properties in terms of clonogenicity, proliferative potential and multipotency. In conditions yet to be defined, the slow-cycling FPC undergo asymmetrical division producing the TA cells, which are responsible for the propagation of progenies.

## Conclusions

In conclusion, we demonstrated a candidate population of endometrial stromal cells with characteristics of slow-cycling and exhibiting common hallmark properties of somatic stem cells. The established *in vitro* method provides an alternate platform for future analyses of putative endometrial stromal stem/progenitor cells. It can also be used for isolating putative somatic stem/progenitor cells in other adult tissues which do not have defined surface marker(s). The existence of endometrial stem/progenitor cells residing in inactive and postmenopausal [3] endometrium raises the possibility that these cells are slow-cycling. The current established technique can be used for the isolation of slow-cycling cells from women with irregular or no cycles, so the molecular mechanisms involved in the self-renewal of endometrial stem/progenitor cells can be elucidated. Furthermore, application of the FPC technique in characterizing the putative endometrial epithelial stem/progenitor cells may be of use, as there has been limited development within this field.

## Abbreviations

AU: absorbance unit; BM: bone marrow; BSA: bovine serum albumin; C/EBPα: CCAAT-enhancer-binding protein α; CBFA1: core binding factor 1; Cdt1-RFP: CdC 10 dependent transcript 1 red fluorescent protein; CFU: colony-forming units; COL2A1: collagen type II alpha 1; (D)MEM: (Dulbecco’s) modified Eagle’s medium; eMSC: endometrial mesenchymal stem cells; FPC: fluorescent persistent cells; IgG: immunoglobulin G; PBS: phosphate-buffered saline; PDGF: platelet-derived growth factor; PI: propidium iodide; PE: phycoerythrin; PPARγ: peroxisome proliferation activated receptor γ; qPCR: quantitative real-time polymerase chain reaction; TA: transit-amplifying; SEM: standard error of the mean; αSMA: alpha smooth muscle actin.

## Competing interests

The authors declare that they have no competing interests.

## Authors’ contributions

LX contributed substantially to the acquisition of the experimental work, analysis and drafting of the manuscript. RC designed the study, performed experimental work and article revisions. EN coordinated the collection of clinical samples and approved the final draft for publication. WY directed the study, obtained financial support for the research and critically revised the final version of the manuscript. All authors read and approved the final manuscript.

## Supplementary Material

Additional file 1: Figure S1 Gating strategy for flow-cytometry sorting of fluorescence persisting cells. Dot plot setup for **(A)** exclusion of debris from live cells based on the Forward Scatter (FSC) and Side Scatter (SSC) plot. **(B)** Cell properties; SSC area (SSC-A) versus SSC height (SSC-H), to gate out cell doublets and aggregates and ensure the signal arises from single cell. Qtracker® dye was recognized by allophycocyanin (APC) laser. Single parameter histogram for **(C)** positive control using freshly stained cells (same gate set approximately 98% for APC, right) and **(D)** negative control using unstained cells (gate set 0% for APC, right). **(E)** Cells were sorted using the gating for isolation of the two populations: left for fluorescence^˗^ (non-FPC) and right for fluorescence^+^ (FPC) expression. Click here for file

Additional file 2: Figure S2 Different patterns of G_1_ phase within a period of 42 hours. The changes of Cdt1 red fluorescence protein in endometrial stromal cells; **(A)** appearance and disappearance within the recording period, **(B)** continuous, **(C)** fluorescence seen at the beginning or **(D)** fluorescence still observed at the end of the recording. Images are representative of one experiment. Click here for file

Additional file 3: Table S1 Induction for differentiation of mesenchymal lineages and the detection of specific markers using histochemical staining, immunofluorescence and real-time PCR. Click here for file

Additional file 4: Table S2 List of primary and secretory antibodies used for immunohistochemistry (IHC) and immunofluorescent (IF) staining. Click here for file

Additional file 5: Table S3 List of primary and secondary antibodies used for western blotting. Click here for file

Additional file 6: Table S4 Pluripotent and self-renewal genes. List of Taqman probes used for real-time PCR. Click here for file

Additional file 7: Figure S3 Differentiation potential of human endometrial stromal FPC and non-FPC into adipogenic lineage. Adipogenic differentiation on cells clonally derived from stromal large CFUs of FPC and non-FPC. Relative gene expression level of C/EBPα. mRNA expression levels were normalized to 18X. Expression of control was set as one. Control is unselected stromal cells grown in culture medium with fetal bovine serum for four weeks. Results are reported as mean ± SEM (n = 6), *P <0.05. Abbreviation: C/EBPα, CCAAT-enhancer-binding protein α. Click here for file

## References

[B1] Vaananen HK Mesenchymal stem cells Ann Med 2005 37 469 479 16278160 10.1080/07853890500371957

[B2] Ferenczy A Bergeron C Histology of the human endometrium: from birth to senescence Ann N Y Acad Sci 1991 622 6 27 2064209 10.1111/j.1749-6632.1991.tb37847.x

[B3] Gargett C Nguyen HT Ye L Endometrial regeneration and endometrial stem/progenitor cells Rev Endocr Metab Disord 2012 13 235 251 22847235 10.1007/s11154-012-9221-9

[B4] Schwab KE Gargett CE Co-expression of two perivascular cell markers isolates mesenchymal stem-like cells from human endometrium Hum Reprod 2007 22 2903 2911 17872908 10.1093/humrep/dem265

[B5] Spitzer TL Rojas A Zelenko Z Aghajanova L Erikson DW Barragan F Meyer M Tamaresis JS Hamilton AE Irwin JC Giudice LC Perivascular human endometrial mesenchymal stem cells express pathways relevant to self-renewal, lineage specification, and functional phenotype Biol Reprod 2012 86 58 22075475 10.1095/biolreprod.111.095885 PMC3290674

[B6] Taylor HS Endometrial cells derived from donor stem cells in bone marrow transplant recipients JAMA 2004 292 81 85 15238594 10.1001/jama.292.1.81

[B7] Du H Taylor HS Contribution of bone marrow-derived stem cells to endometrium and endometriosis Stem Cells 2007 25 2082 2086 17464086 10.1634/stemcells.2006-0828

[B8] Mikkers H Frisen J Deconstructing stemness EMBO J 2005 24 2715 2719 16037819 10.1038/sj.emboj.7600749 PMC1182244

[B9] Neumuller RA Knoblich JA Dividing cellular asymmetry: asymmetric cell division and its implications for stem cells and cancer Genes Dev 2009 23 2675 2699 19952104 10.1101/gad.1850809 PMC2788323

[B10] Snippert HJ Clevers H Tracking adult stem cells EMBO Rep 2011 12 113 122 21252944 10.1038/embor.2010.216 PMC3049439

[B11] Li L Clevers H Coexistence of quiescent and active adult stem cells in mammals Science 2010 327 542 545 20110496 10.1126/science.1180794 PMC4105182

[B12] Grompe M Tissue stem cells: new tools and functional diversity Cell Stem Cell 2012 10 685 689 22704508 10.1016/j.stem.2012.04.006 PMC3940056

[B13] Chan RW Gargett CE Identification of label-retaining cells in mouse endometrium Stem Cells 2006 24 1529 1538 16456137 10.1634/stemcells.2005-0411

[B14] Kaitu'u-Lino TJ Ye L Salamonsen LA Girling JE Gargett CE Identification of label-retaining perivascular cells in a mouse model of endometrial decidualization, breakdown, and repair Biol Reprod 2012 86 184 22402967 10.1095/biolreprod.112.099309

[B15] Patterson AL Pru JK Long-term label retaining cells localize to distinct regions within the female reproductive epithelium Cell Cycle 2013 12 2888 2898 24018418 10.4161/cc.25917 PMC3899201

[B16] Seleverstov O Zabirnyk O Zscharnack M Bulavina L Nowicki M Heinrich JM Yezhelyev M Emmrich F O'Regan R Bader A Quantum dots for human mesenchymal stem cells labeling. A size-dependent autophagy activation Nano Lett 2006 6 2826 2832 17163713 10.1021/nl0619711

[B17] Danner S Benzin H Vollbrandt T Oder J Richter A Kruse C Quantum dots do not alter the differentiation potential of pancreatic stem cells and are distributed randomly among daughter cells Int J Cell Biol 2013 2013 918242 23997768 10.1155/2013/918242 PMC3742022

[B18] Noyes RW Hertig AT Rock J Dating the endometrial biopsy Am J Obstet Gynecol 1975 122 262 263 1155504 10.1016/s0002-9378(16)33500-1

[B19] Chan RW Schwab KE Gargett CE Clonogenicity of human endometrial epithelial and stromal cells Biol Reprod 2004 70 1738 1750 14766732 10.1095/biolreprod.103.024109

[B20] Sakaue-Sawano A Kurokawa H Morimura T Hanyu A Hama H Osawa H Kashiwagi S Fukami K Miyata T Miyoshi H Imamura T Ogawa M Masai H Miyawaki A Visualizing spatiotemporal dynamics of multicellular cell-cycle progression Cell 2008 132 487 498 18267078 10.1016/j.cell.2007.12.033

[B21] Chan RW Ng EH Yeung WS Identification of cells with colony-forming activity, self-renewal capacity, and multipotency in ovarian endometriosis Am J Pathol 2011 178 2832 2844 21641404 10.1016/j.ajpath.2011.02.025 PMC3123988

[B22] Livak KJ Schmittgen TD Analysis of relative gene expression data using real-time quantitative PCR and the 2(−delta delta C(T)) method Methods 2001 25 402 408 11846609 10.1006/meth.2001.1262

[B23] Masuda H Anwar SS Buhring HJ Rao JR Gargett CE A novel marker of human endometrial mesenchymal stem-like cells Cell Transplant 2012 21 2201 2214 22469435 10.3727/096368911X637362

[B24] Fafet P Rebouissou C Maudelonde T Vignais ML Opposite effects of transforming growth factor-beta activation and rho-associated kinase inhibition on human trophoblast migration in a reconstituted placental-endometrial coculture system Endocrinology 2008 149 4475 4485 18499753 10.1210/en.2008-0253

[B25] Kashyap V Rezende NC Scotland KB Shaffer SM Persson JL Gudas LJ Mongan NP Regulation of stem cell pluripotency and differentiation involves a mutual regulatory circuit of the NANOG, OCT4, and SOX2 pluripotency transcription factors with polycomb repressive complexes and stem cell microRNAs Stem Cells Dev 2009 18 1093 1108 19480567 10.1089/scd.2009.0113 PMC3135180

[B26] Lukacs RU Memarzadeh S Wu H Witte ON Bmi-1 is a crucial regulator of prostate stem cell self-renewal and malignant transformation Cell Stem Cell 2010 7 682 693 21112563 10.1016/j.stem.2010.11.013 PMC3019762

[B27] Masuda H Matsuzaki Y Hiratsu E Ono M Nagashima T Kajitani T Arase T Oda H Uchida H Asada H Ito M Yoshimura Y Maruyama T Okano H Stem cell-like properties of the endometrial side population: implication in endometrial regeneration PLoS One 2010 5 e10387 20442847 10.1371/journal.pone.0010387 PMC2860997

[B28] Cervelló I Gil-Sanchis C Mas A Delgado-Rosas F Martínez-Conejero JA Galán A Martínez-Romero A Martínez S Navarro I Ferro J Horcajadas JA Esteban FJ O'Connor JE Pellicer A Simon C Human endometrial side population cells exhibit genotypic, phenotypic and functional features of somatic stem cells PLoS One 2010 5 e10964 20585575 10.1371/journal.pone.0010964 PMC2891991

[B29] Lee GM Fong SS Oh DJ Francis K Palsson BO Characterization and efficacy of PKH26 as a probe to study the replication history of the human hematopoietic KG1a progenitor cell line In Vitro Cell Dev Biol Anim 2002 38 90 96 11929001 10.1290/1071-2690(2002)038<0090:CAEOPA>2.0.CO;2

[B30] Dembinski JL Krauss S Characterization and functional analysis of a slow cycling stem cell-like subpopulation in pancreas adenocarcinoma Clin Exp Metastasis 2009 26 611 623 19421880 10.1007/s10585-009-9260-0 PMC2776152

[B31] Reynolds BA Rietze RL Neural stem cells and neurospheres–re-evaluating the relationship Nat Methods 2005 2 333 336 15846359 10.1038/nmeth758

[B32] Orford KW Scadden DT Deconstructing stem cell self-renewal: genetic insights into cell-cycle regulation Nat Rev Genet 2008 9 115 128 18202695 10.1038/nrg2269

[B33] Rosen ED Walkey CJ Puigserver P Spiegelman BM Transcriptional regulation of adipogenesis Genes Dev 2000 14 1293 1307 10837022

[B34] Rajaraman G White J Tan KS Ulrich D Rosamilia A Werkmeister J Gargett CE Optimization and scale-up culture of human endometrial multipotent mesenchymal stromal cells: potential for clinical application Tissue Eng Part C Methods 2012 19 80 92 22738377 10.1089/ten.tec.2011.0718 PMC3522126

[B35] Sivasubramaniyan K Harichandan A Schumann S Sobiesiak M Lengerke C Maurer A Kalbacher H Buhring HJ Prospective isolation of mesenchymal stem cells from human bone marrow using novel antibodies directed against Sushi domain containing 2 Stem Cells Dev 2013 22 1944 1954 23406305 10.1089/scd.2012.0584

[B36] Liu CM Yu CH Chang CH Hsu CC Huang LL Hyaluronan substratum holds mesenchymal stem cells in slow-cycling mode by prolonging G1 phase Cell Tissue Res 2008 334 435 443 18953571 10.1007/s00441-008-0699-0

[B37] Simon C Götz M Dimou L Progenitors in the adult cerebral cortex: cell cycle properties and regulation by physiological stimuli and injury Glia 2011 59 869 881 21446038 10.1002/glia.21156

[B38] Roccio M Schmitter D Knobloch M Okawa Y Sage D Lutolf MP Predicting stem cell fate changes by differential cell cycle progression patterns Development 2013 140 459 470 23193167 10.1242/dev.086215

[B39] Lange C Calegari F Cdks and cyclins link G1 length and differentiation of embryonic, neural and hematopoietic stem cells Cell Cycle 2010 9 1893 1900 20436288 10.4161/cc.9.10.11598

[B40] Nygren JM Bryder D Jacobsen SE Prolonged cell cycle transit is a defining and developmentally conserved hemopoietic stem cell property J Immunol 2006 177 201 208 16785515 10.4049/jimmunol.177.1.201

[B41] Salamonsen L Shuster S Stern R Distribution of hyaluronan in human endometrium across the menstrual cycle Cell Tissue Res 2001 306 335 340 11702245 10.1007/s004410100452

