# Supplementary figures and images for: Nanoparticle labeling identifies slow cycling human endometrial stromal cells
Source: Stem Cell Res Ther. 2014 Jul 4;5(4):84. doi: 10.1186/scrt473 (PMC4230801; doi:10.1186/scrt473)

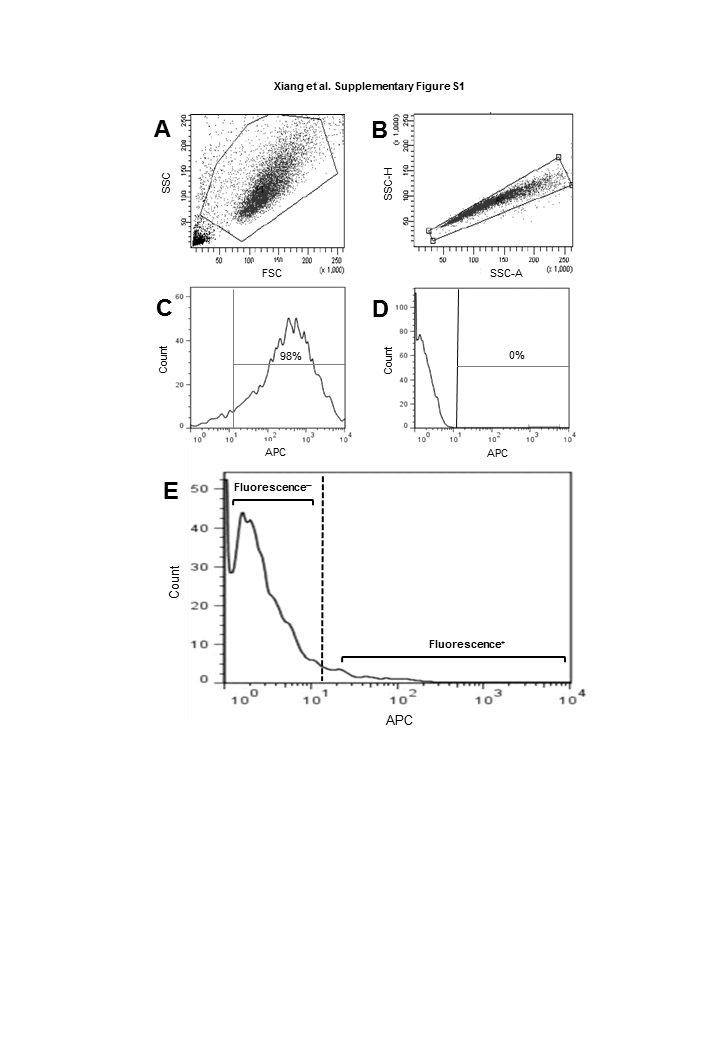

Supplement: Additional file 1: Figure S1 — Gating strategy for flow-cytometry sorting of fluorescence persisting cells. Dot plot setup for (A) exclusion of debris from live cells based on the Forward Scatter (FSC) and Side Scatter (SSC) plot. (B) Cell properties; SSC area (SSC-A) versus SSC height (SSC-H), to gate out cell doublets and aggregates and ensure the signal arises from single cell. Qtracker® dye was recognized by allophycocyanin (APC) laser. Single parameter histogram for (C) positive control using freshly stained cells (same gate set approximately 98% for APC, right) and (D) negative control using unstained cells (gate set 0% for APC, right). (E) Cells were sorted using the gating for isolation of the two populations: left for fluorescence˗ (non-FPC) and right for fluorescence+ (FPC) expression. [file scrt473-S1.doc]

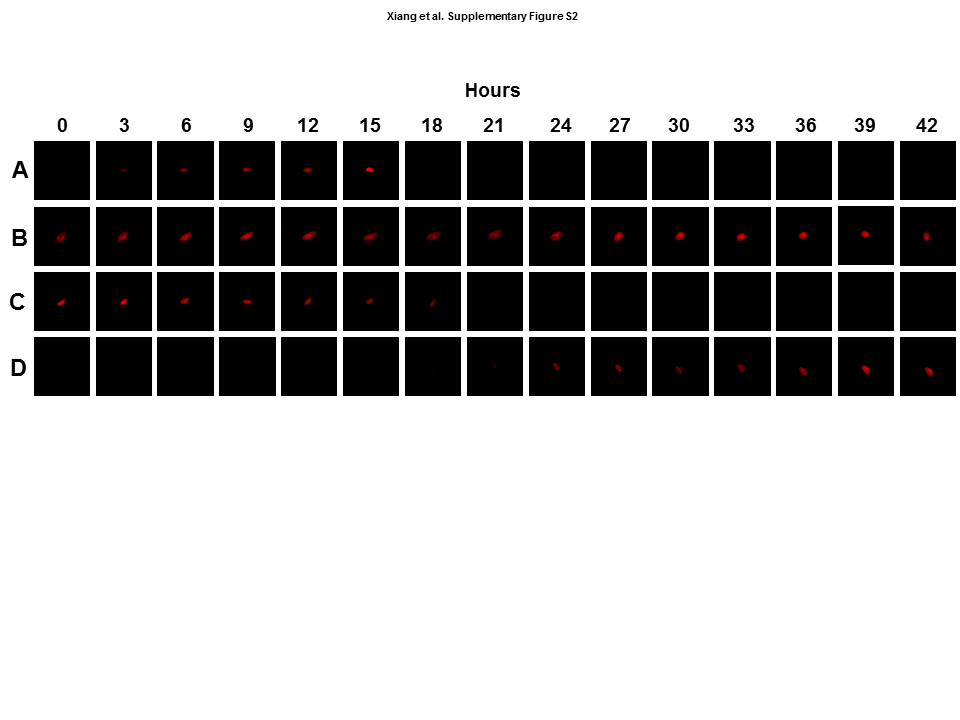

Supplement: Additional file 2: Figure S2 — Different patterns of G1 phase within a period of 42 hours. The changes of Cdt1 red fluorescence protein in endometrial stromal cells; (A) appearance and disappearance within the recording period, (B) continuous, (C) fluorescence seen at the beginning or (D) fluorescence still observed at the end of the recording. Images are representative of one experiment. [file scrt473-S2.doc]

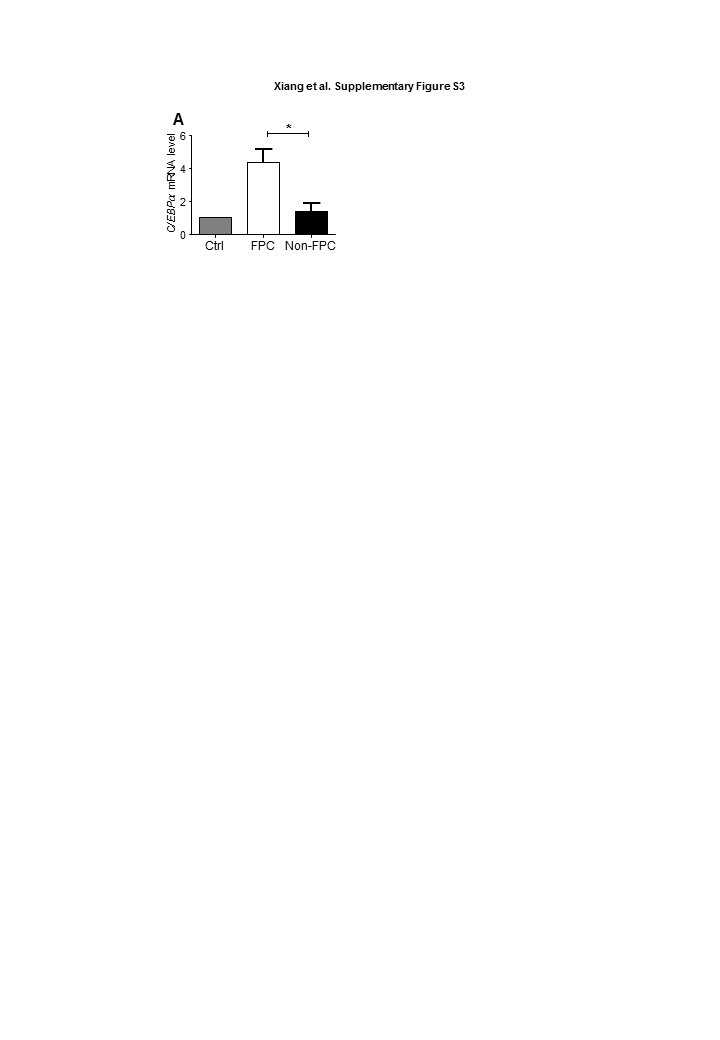

Supplement: Additional file 7: Figure S3 — Differentiation potential of human endometrial stromal FPC and non-FPC into adipogenic lineage. Adipogenic differentiation on cells clonally derived from stromal large CFUs of FPC and non-FPC. Relative gene expression level of C/EBPα. mRNA expression levels were normalized to 18X. Expression of control was set as one. Control is unselected stromal cells grown in culture medium with fetal bovine serum for four weeks. Results are reported as mean ± SEM (n = 6), *P <0.05. Abbreviation: C/EBPα, CCAAT-enhancer-binding protein α. [file scrt473-S7.doc]
