# Supplementary material for: Nanoparticle labeling identifies slow cycling human endometrial stromal cells
Source: Stem Cell Res Ther. 2014 Jul 4;5(4):84. doi: 10.1186/scrt473 (PMC4230801; doi:10.1186/scrt473)
Supplement: Additional file 3: Table S1 — Induction for differentiation of mesenchymal lineages and the detection of specific markers using histochemical staining, immunofluorescence and real-time PCR. [file scrt473-S3.doc]

**Xiang et al. Additional file 3: Table S1**

**Additional file 3: Table S1- Induction for differentiation of mesenchymal lineages and the detection of specific markers using histochemical staining, immunofluorescence and real-time PCR**

| **Lineage** | **Induction Media Components** | **Staining** | **Taqman Probe** |
| --- | --- | --- | --- |
| Myogenic | DMEM F12 Medium, FBS,  50 µM Hydrocortisone,  Penicillin | α Smooth Muscle Actin (αSMA). | Alpha Actin 2 *(ACTA2)*  Hs00426835_g1 |
| Osteogenic | DMEM F12 Medium, FBS,  0.01µM 1,25-dihydroxyvitamin D3  50µM Ascorbate-2-phosphate,  10 mM β-glycerophosphate,  Penicillin | Osteopontin | Core-Binding Factor Alpha Subunit 1 (*CBFA1*)  Hs00231692 |
|  |  |  | Osteopontin (*OPN*)  Hs00959010_m1 |
| Chondrogenic | DMEM F12 Medium, FBS  6.25ug/mL Insulin,  10 ng/mL TGF-β1,  50μM Ascorbate-2-phosphate, Penicillin | Safranin-O,  Collagen Type II. | Collagen II (COL2AI)  Hs00264051_m1 |
| Adipogenic | DMEM F12 Medium, FBS  0.5mM Isobutyl-methylxanthine,  1 µM Dexamethasone,  10 µM Insulin,  200μM Indomethacin,  Penicillin | Oil Red O,  Peroxisome Proliferation Activated Receptor γ (PPARγ). | CCA-AT/Enhancer Binding Protein alpha (*C/EBPα*)  Hs00269972_s1 |
