# Supplementary material for: Nanoparticle labeling identifies slow cycling human endometrial stromal cells
Source: Stem Cell Res Ther. 2014 Jul 4;5(4):84. doi: 10.1186/scrt473 (PMC4230801; doi:10.1186/scrt473)
Supplement: Additional file 4: Table S2 — List of primary and secretory antibodies used for immunohistochemistry (IHC) and immunofluorescent (IF) staining. [file scrt473-S4.doc]

**Xiang et al. Additional file 4: Table S2**

**Additional file 4: Table S2- List of primary and secretory antibodies used for immunohistochemistry (IHC) and immunofluorescent (IF) staining.**

| **Primary Antibodies** | **Dilution** | **Source** |
| --- | --- | --- |
| **αSMA:** mouse monoclonal α smooth muscle actin; clone 1A4. | 1:300 | Dako |
| **Osteopontin:** rabbit polyclonal to osteopontin. | 1:500 | Abcam |
| **PPARγ:**  rabbit Peroxisome proliferators-activated receptor γ; clone C26H12. | 1:1000 | Cell Signaling |
| **Collagen II:** rabbit polyclonal to collagen II. | 1:500 | Abcam |
| **Secondary Antibodies** | **Dilution** | **Source** |
| Rabbit-anti-mouse (IHC) | 1:200 | Dako |
| Goat-anti-rabbit (IHC) | 1:200 | Dako |
| Alex Fluor 568 Donkey anti-rabbit (IF; Red Fluorchrome) | 1:500 | Invitrogen |
