# Supplementary material for: Nanoparticle labeling identifies slow cycling human endometrial stromal cells
Source: Stem Cell Res Ther. 2014 Jul 4;5(4):84. doi: 10.1186/scrt473 (PMC4230801; doi:10.1186/scrt473)
Supplement: Additional file 5: Table S3 — List of primary and secondary antibodies used for western blotting. [file scrt473-S5.doc]

**Xiang et al. Additional file 5: Table S3**

**Additional file 5:** Table S3 - List of primary and secondary antibodies used for western blotting

| **Primary Antibodies** | | **Isotype** | | **Dilution** | **Source** |
| --- | --- | --- | --- | --- | --- |
| **αSMA:** mouse monoclonal α smooth muscle actin; clone 1A4. | | Mouse IgG2a | | 1:1000 | Dako |
| **Osteopontin:** rabbit polyclonal to osteopontin. | | Rabbit IgG | | 1:1000 | Abcam |
| **Collagen II:** rabbit polyclonal to collagen II. | | Rabbit IgG | | 1:1000 | Abcam |
| **PPARγ:** rabbit Peroxisome proliferators-activated receptor γ; clone C26H12. | | Rabbit IgG | | 1:1000 | Cell Signaling |
| **β-actin**: mouse monoclonal beta actin; clone AC-15. | | Mouse IgG1 | | 1:10000 | Sigma |
| **Secondary Antibodies** | **Dilution** | | **Source** | | |
| Sheep Anti-Mouse IgG | 1:5000 | | GE Healthcare | | |
| Donkey Anti-Rabbit IgG | 1:5000 | | GE Healthcare | | |
