# Supplementary material for: Nanoparticle labeling identifies slow cycling human endometrial stromal cells
Source: Stem Cell Res Ther. 2014 Jul 4;5(4):84. doi: 10.1186/scrt473 (PMC4230801; doi:10.1186/scrt473)
Supplement: Additional file 6: Table S4 — Pluripotent and self-renewal genes. List of Taqman probes used for real-time PCR. [file scrt473-S6.doc]

**Xiang et al. Additional file 6: Table S4**

**Additional file 6:** Table S4 – Pluripotent and self-renewal genes. List of Taqman probes used for real-time PCR.

| **Taqman Probe** |
| --- |
| **NANOG:** Hs04260366_g1 |
| **SOX-2:** Hs01053049_S1 |
| **OCT-4:** Hs00742896_s1 |
| **BMI-1:** Hs00180411_m1 |
